# Translating Evidence to Advanced Parkinson's Disease Patients: A Systematic Review and Meta‐Analysis

**DOI:** 10.1002/mds.28599

**Published:** 2021-04-02

**Authors:** Frouke A.P. Nijhuis, Rianne Esselink, Rob M.A. de Bie, Hans Groenewoud, Bastiaan R. Bloem, Bart Post, Marjan J. Meinders

**Affiliations:** ^1^ Department of Neurology Canisius Wilhelmina Hospital Nijmegen the Netherlands; ^2^ Department of Neurology Radboud University Medical Center, Donders Institute for Brain, Cognition and Behavior Nijmegen the Netherlands; ^3^ Department of Neurology Amsterdam University Medical Centers, University of Amsterdam, Amsterdam Neuroscience Amsterdam the Netherlands; ^4^ Department for Health Evidence Radboud University Medical Center Nijmegen the Netherlands

**Keywords:** advanced PD therapies, systematic review, Parkinson's disease, patient‐relevant outcomes, evidence‐based medicine

## Abstract

In the advanced stages of Parkinson's disease (PD), patients frequently experience disabling motor complications. Treatment options include deep brain stimulation (DBS), levodopa‐carbidopa intestinal gel (LCIG), and continuous subcutaneous apomorphine infusion (CSAI). Choosing among these treatments is influenced by scientific evidence, clinical expertise, and patient preferences. To foster patient engagement in decision‐making among the options, scientific evidence should be adjusted to their information needs. We conducted a systematic review from the patient perspective. First, patients selected outcomes for a treatment choice: quality of life, activities of daily living, ON and OFF time, and adverse events. Second, we conducted a systematic review and meta‐analysis for each treatment versus best medical treatment using Grading of Recommendations, Assessment, Development, and Evaluation (GRADE). Finally, the evidence was transformed into comprehensible and comparable information. We converted the meta‐analysis results into the number of patients (per 100) who benefit clinically from an advanced treatment per outcome, based on the minimal clinically important difference and the cumulative distribution function. Although this approach allows for a comparison of outcomes across the three device‐aided therapies, they have never been compared directly. The interpretation is hindered by the relatively short follow‐up time in the included studies, usually less than 12 months. These limitations should be clarified to patients during the decision‐making process. This review can help patients integrate the evidence with their own preferences, and with their clinician's expertise, to reach an informed decision. © 2021 The Authors. *Movement Disorders* published by Wiley Periodicals LLC on behalf of International Parkinson and Movement Disorder Society

In the advanced stages of Parkinson's disease (PD), most patients develop motor complications, which can have a significant impact on quality of life.^1^ For many patients, it is difficult to manage these complications with oral medication. There are three advanced treatments available: deep brain stimulation (DBS), levodopa‐carbidopa intestinal gel (LCIG), and continuous subcutaneous apomorphine infusion (CSAI). The choice of one of these treatments versus continuing best medical treatment (BMT) should be based ideally on combined input from scientific evidence, clinical expertise, and individual patient preferences,^2^ likely supplemented by big data analyses.^3^

In our previous study, patients reported that not all options were discussed.^4^ Several factors contributed: patient ineligibility for a treatment, physician treatment preference, and limited treatment availability. Patient characteristics (mild cognitive impairment, comorbidities, independence) often guide physician treatment choice. However, a large group of patients is still eligible for all treatments, and patient preferences should be included in decision‐making. Patients can only construct their preferences if they know all the treatment options and are aware of their benefits and risks. They need balanced, objective, and reliable information based on the best available evidence to facilitate this complex information exchange and decision‐making. In the case of advanced PD treatments, the evidence is not readily available for direct comparison of all treatments.^5^, ^6^ Furthermore, framing of benefits and risks of treatment options can influence patient choice.^7^ The format for presenting the possible benefits and risks is therefore important.

The aim of this systematic review and meta‐analysis was two‐fold: (1) to assess the scientific evidence about effectiveness of advanced treatments for outcomes that PD patients find crucial for their treatment choice; and (2) to translate the evidence into comprehensible information formats.

## 1 Methods

### 1.1 Selection of Relevant Outcome Measures

We used data obtained from our previous studies^4^, ^8^ to select relevant outcomes. We first organized six focus groups and three interviews with 16 caregivers and 20 patients who had previously received an advanced treatment.^8^ This produced a list of items that patients and caregivers regarded as relevant when choosing an advanced treatment. Next, 111 patients prioritized these items in a survey.^4^ Patients prioritized the following as most important: quality of life (QoL), activities of daily living (ADL), physical effects, and complications/adverse effects. For QoL, we used the Parkinson's Disease Questionnaire (PDQ‐39) or PDQ‐8 scales. For ADL we chose the Unified Parkinson's Disease Rating Scale Part II (UPDRS II). OFF time and ON time, as a representation of physical effects, were expressed in hours. We defined a good ON time as ON time without troublesome dyskinesia. Most controlled studies have used these outcomes.

### 1.2 Literature Search

We conducted a systematic literature search from inception of each database to February 2019 and updated the search monthly until May 2020. The questions addressed were: (1) what is the effect of the interventions (DBS, LCIG, and CSAI) compared to each other on each outcome (QoL, ADL, ON and OFF time, and complications/adverse effects) in advanced PD patients; and (2) what is the effect of each intervention compared to BMT on each outcome in these patients. The search covered PubMed, EMBASE, and Cochrane Central Register of Controlled Trials and was supplemented with hand searches in reference lists of included studies. The search strategy, developed by an independent and experienced librarian, included a range of keywords (both MeSH and free text): DBS, deep brain stimulation, subthalamic nucleus stimulation, global pallidus stimulation, Duodopa, levodopa‐carbidopa intestinal gel, LCIG, intestinal levodopa, continuous apomorphine infusion, CSAI, or related synonyms. The search strategy is available upon request.

### 1.3 Study Selection

Two independent researchers (F.A.P.N., P.P.R.) screened all titles/abstracts based on inclusion and exclusion criteria. In case of disagreement, the final decision was based on the full‐text article. The inclusion criteria were: (1) the study population consists of advanced PD patients with severe motor fluctuations and/or dyskinesias despite BMT; (2) the study includes at least one outcome measure of our interest (QoL, ADL, ON or OFF time, adverse events); (3) the study either directly compares all three treatments (CSAI, DBS, and LCIG) or compares one of the treatments (CSAI, DBS, or LCIG) to BMT; (4) the study includes at least 10 participants; and (5) the study is a randomized controlled trial (RCT), prospective non‐RCT, or prospective cohort study. The definition of advanced PD is not straightforward but includes some key indicators of both motor and non‐motor functions. Motor functions include moderately troublesome motor fluctuations, at least 1 hour of troublesome dyskinesia/day, at least 2 hours of OFF symptoms/day, and at least five oral levodopa doses/day.^9^ Exclusion criteria were: (1) the study compares different DBS targets, unless it also compares DBS to BMT; (2) the study concerns stimulation of other than the subthalamic or pallidal nuclei; (3) the study compares an intervention with placebo instead of BMT, unless the placebo group also continues BMT; (4) the study compares an intervention with oral levodopa alone and not BMT; and (5) the study is a retrospective study. Only full‐text articles were considered for inclusion.

The full‐text articles of all selected abstracts were assessed independently by two researchers (F.A.P.N., W.D.) using a data collection form. In case of disagreement between reviewers, a third independent reviewer (B.P.) was consulted, and consensus was reached through discussion.

For each comparison (CSAI vs. DBS vs. LCIG, CSAI vs. BMT, DBS vs. BMT, LCIG vs. BMT) the quality and certainty of evidence was determined using the Grading of Recommendations, Assessment, Development, and Evaluation (GRADE) approach.^10^ Since a very small number of studies met all criteria for some of the treatments or outcomes, non‐randomized studies were also included when no RCTs were available or the level of certainty from the RCTs was low or very low.

### 1.4 Data Extraction and Quality Assessment

One reviewer (F.A.P.N.) extracted data on study design, patient population (inclusion and exclusion criteria, number of patients, duration of disease, duration of levodopa treatment, concomitant treatment), type of intervention (CSAI, DBS, LCIG), intervention procedures (type of operation, dose levels), length of follow‐up, number of patients lost to follow‐up, and outcome measures.

One reviewer (F.A.P.N.) assessed the included studies for methodological quality and discussed this with a small review team (F.A.P.N., M.J.M., B.P.) using GRADE.^10^ Specifically, we assessed the study limitations by evaluating randomization method, allocation concealment, blinding method, intention to treat analysis, and loss to follow‐up data. As per GRADE protocol, we also assessed the certainty of evidence of inconsistency (heterogeneity), indirectness, imprecision, and other potential sources of bias, including publication bias. The GRADE criteria were then applied to downgrade the certainty of evidence per specific outcome of each comparison. The certainty of evidence for an individual outcome was ultimately rated as high, moderate, low, or very low.

### 1.5 Data Synthesis and Analysis

In case at least two studies addressed the same outcome, a meta‐analysis was used to compute a pooled score. Depending on the character of the outcome measure, either pooled mean difference or pooled proportion was computed. In case only one study reported results for an outcome measure, the point estimate of that study was used. Heterogeneity was assessed using the Tau^2^ and I^2^ statistic. Tau^2^ is defined as the variance of the true effect sizes, and the I^2^ describes the percentage of variation across studies that is due to heterogeneity rather than chance.^11^, ^12^ Because considerable heterogeneity was present in several studies, we always used a random effect model to compute the pooled score. In case large heterogeneity occurred, the possible causes were studied.

### 1.6 Transformation to Clinical Practice

**FIG. 1:**
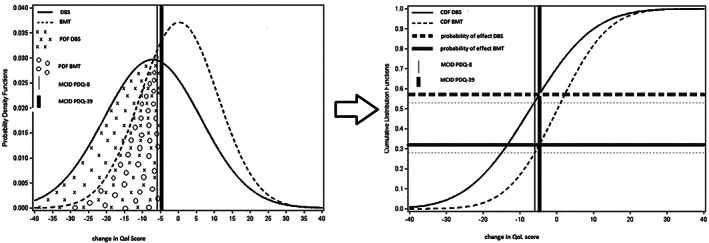
Calculation of probability of clinically relevant effect for quality of life in deep brain stimulation (DBS) versus best medical treatment (BMT). PDF, probability density function; QoL, quality of life; MCID, minimal clinically important difference; PDQ, Parkinson's Disease Questionnaire; CDF, cumulative distribution function.

To translate the outcomes into a plain language format, we calculated the chance of success of a treatment. The chance of success is defined as the probability of realizing a positive result equal or more than the minimal clinically important difference (MCID) of an outcome measure. The probability of a clinically relevant effect is expressed in number of patients who have a clinically relevant effect when 100 patients are treated. The probability density function reflects the probability of the random variable falling within a particular range of values. It is given by the area under the density function and between the lowest and greatest values of the chosen range (Fig. 1). We selected the MCID for each outcome from the literature.^13^, ^14^, ^15^ For most scales (PDQ‐39, PDQ‐8, UPDRS II, and OFF time), improvement meant a lower score, and therefore the MCID is negative. For ON time, improvement is shown as an increase in hours, and therefore the MCID is positive. Assuming outcomes follow a normal distribution with a given mean and standard deviation, the cumulative distribution function (CDF) can be used to calculate the probability of a clinical relevant improvement (Fig. 1).^16^ For example, Fig. 1 represents the normal distribution curve of the mean difference effects in QoL for the DBS group (continuous line) and the BMT group (dotted line). The cumulative distribution function is the probability that a variable takes a value ≤X, in which X represents the MCID. In the example of QoL that is −4.72 for PDQ‐39 (thick vertical black line) and −5.94 for PDQ‐8 (vertical grey line). The probability of a clinically relevant effect in QoL (PDQ‐39) for DBS and BMT can then be deducted from the left graph (Fig. 1). This was done for all outcomes. For ON time duration, the CDF was corrected for the positive MCID.

### 1.7 Data Availability Policy

All data included in this review are available in the articles indicated in Table 1. The list of most important excluded studies based on full‐text analysis, including the reason for exclusion, are available on request. All GRADE reviews and details can be provided upon request. This review follows the Preferred Reporting Items for Systematic Reviews and Meta‐Analyses (PRISMA) criteria for reporting.^17^

**TABLE 1:** Characteristics of the included studies

Study and year	Study design	Follow up (SD)[range]	Participants in group (n)	Outcomes	Notes
**Intervention: all advanced treatments**					
Dafsari 2019	Prospective, non‐randomized, open‐label multicenter, real‐life cohort study of CSAI, DBS, LCIG	6 mo	39(CSAI)/101 (DBS)/33(LCIG)	QoL, adverse events	Large group of real‐life cohort study, therefore not comparable groups at baseline
**Intervention: DBS original RCTs (additional articles of same RCT)**					
Deuschl 2006 (Witt 2008, Daniels 2011, Witt 2011)	Randomized (paired), controlled, unblinded trial DBS vs. BMT	6 mo	78 (DBS)/78 (BMT)	QoL, ADL ON/OFF time, adverse events	
Schuepbach 2007 (Lhommee 2018, Schuepbach 2019)	Randomized (matched pairs), controlled, unblinded trial (pilot study)	18 mo	10 (DBS)/10 (BMT)	QoL, ADL	Patients with shorter disease duration with motor fluctuations
Weaver 2009 (Weaver 2012, Rothlind 2015)	Randomized, controlled, multicenter trial Blinded motor assessment stratification by study site and age	6 mo	121 (DBS)/134 (BMT)	ADL, ON/OFF time, adverse events	
Williams 2010	Randomized (pairwise), unblinded trial	1 y	183(DBS)/183(BMT)	QoL, ADL, OFF time, adverse events	Over 1/3 used apomorphine as well, unclear if this was CSAI or injections
Schuepbach 2013	Randomized, controlled, multicenter, unblinded trial	24 mo	124 (DBS)/127 (BMT)	QoL, ADL, ON/OFF time, adverse events	Patients with shorter disease duration with motor fluctuations
**Intervention: LCIG original RCTs (additional articles of same RCT)**					
Kurth 1993	Double‐blinded, placebo‐controlled, cross‐over study, randomized in six different treatment schemes of LCIG vs. BMT	4 d	10	ON/OFF time	LCIG (and placebo) through nasoduodenal tube
Nyholm 2005 (Isacson 2008)	Randomized, multicenter, cross‐over trial LCIG vs. BMT	6 wk and 6 mo	25	QoL, ON/OFF time	Only 12 patients available for follow‐up data, LCIG through nasoduodenal tube
Olanow 2014 (Antonini 2016)	Randomized, multicenter, double‐blinded, placebo‐controlled trial of LCIG vs. BMT	12 wk	37 LCIG(+BMT)/34 placebo (+BMT)	QoL, ADL, ON/OFF time	Placebo group also had PEG‐J surgery
**Intervention: LCIG non‐randomized studies/cohort studies (additional articles of same study)**					
Antonini 2008	Prospective, before−after, open‐label, multicenter study of LCIG	2 y	22	QoL, ADL, adverse events	
Palhagen 2012/Palhagen 2016	Prospective, open‐label, multicenter, cohort study of LCIG	3 y	77 (36 LCIG naïve patients)	QoL, ADL, adverse events	Large group already treated with LCIG, in our analysis only LCIG naïve population is taken
Fernandez 2013/Fernandez 2015	Prospective, multicenter, before−after, open‐label study of LCIG	54 wk	354	QoL, ADL, ON/OFF time, adverse events	Fernandez 2013 is interim analysis, Fernandez 2015 is final analysis
Zibetti 2013	Prospective, open‐label, multicenter, cohort study of LCIG	3 y	25	QoL, ADL, adverse events	
Caceres‐Redondo 2014	Prospective, before−after, open‐label study of LCIG	32.2 ± 12.4 mo	29	QoL, ADL, adverse events	
Antonini 2015/Antonini 2017	Prospective, multicenter, before−after, open‐label study of LCIG	24 mo	375 (225 prospective cohort)	QoL, ADL, ON/OFF time, adverse events	Includes retrospective cohort and prospective cohort. We only included the prospective cohort
Bohlega 2015	Prospective, before−after, open‐label study of LCIG	48.5 ± 23.2 mo	20	QoL, adverse events	
Martinez‐Martin 2015	Prospective, non‐randomized, comparative, open‐label study CSAI vs. LCIG (data for before−after study of CSAI and LCIG)	6 mo	44	QoL, adverse events	Stated as a comparative trial CSAI vs. LCIG, however separate before−after data are presented
Slevin 2015	Open‐label, multicenter, extension study of the RCT (Olanow 2014) of LCIG vs. BMT	52 wk	62 (29 naïve)	QoL, ADL, ON/OFF time, adverse events	Patients in BMT group of RCT were offered to switch to LCIG and were analyzed in this study as naive LCIG group alongside the continuous LCIG treatment group (and a combined analysis as well)
Catalan 2018	Prospective, multicenter, before−after, open‐label study of LCIG	6 mo	62	ADL, ON/OFF time	
Ciurleo 2018	Prospective, before−after, open‐label study of LCIG	6 mo	12	QoL	
Vijiaratnam 2018	Prospective, before−after, open‐label study of LCIG	6 mo	25	QoL, ADL, adverse events	
**Intervention: CSAI RCT**					
Katzenschlager 2018	Randomized, placebo‐controlled, double‐blind, multicenter trial of CSAI vs. BMT	12 wk	53(CSAI)/54(BMT)	QoL, ADL, ON/OFF time, adverse events	Only RCT with relevant outcomes for our study
**Intervention: CSAI non‐randomized studies/cohort studies (additional articles of same study)**					
Stibe 1988	Prospective, before−after, open‐label study with 2 groups (CSAI and apomorphine injections)	8 mo [1–15]	11 CSAI	ON/OFF time	In our analysis only CSAI included
Pietz 1998	Prospective, before−after, open‐label study with 2 groups (CSAI and apomorphine injections)	20.2 mo (54.0 mo)	25 CSAI	ADL, OFF time	In our analysis only CSAI included
Kanovsky 2002	Prospective, before−after, open‐label study	2 y	12 CSAI	ON/OFF time, adverse events	
Di Rosa 2003/Morgante 2004	Non‐randomized, open‐label, blinded‐rater, parallel‐group trial CSAI vs. BMT	1 y/2 y	12 (CSAI)/18 (BMT)	OFF time, adverse events	The two articles describe the same study population but different follow‐up. An important exclusion criterion was age above 65 y
Katzenschlager 2005	Prospective, 2 centers before−‐after study, blinded rating of video assessments	6 mo	12 CSAI	ON/OFF time, adverse events	
De Gaspari 2006/Antonini 2011	Prospective, before−after, open‐label study of CSAI (or DBS patients)	1 y 5 y	12 CSAI	OFF time	Antonini study not included for analysis as only 2/13 patients reached 5‐y follow‐up
Martinez‐Martin 2011	Non‐randomized, open‐label, parallel group trial CSAI vs. BMT	12.5 mo (11.5 mo)	17 (CSAI)/17 (BMT)	QoL	No accurate control group, no comparable groups at baseline
Martinez‐Martin 2015	Prospective, non‐randomized, comparative, open‐label study CSAI vs. LCIG (data for before−after study of CSAI and LCIG)	6 mo	43 CSAI	QoL, adverse events	Stated as a comparative trial CSAI vs. LCIG, however separate before−after data are presented

Abbreviations: SD, standard deviation; CSAI, continuous subcutaneous apomorphine infusion; DBS, deep brain stimulation; LCIG, levodopa‐carbidopa intestinal gel; mo, month; QoL, quality of life; RCT, randomized controlled trial; BMT, best medical treatment; ADL, activities of daily living; mo; months; ON/OFF time: duration of on time and duration of OFF time.

## 2 Results

### 2.1 Literature Search and Study Selection

**FIG. 2:**
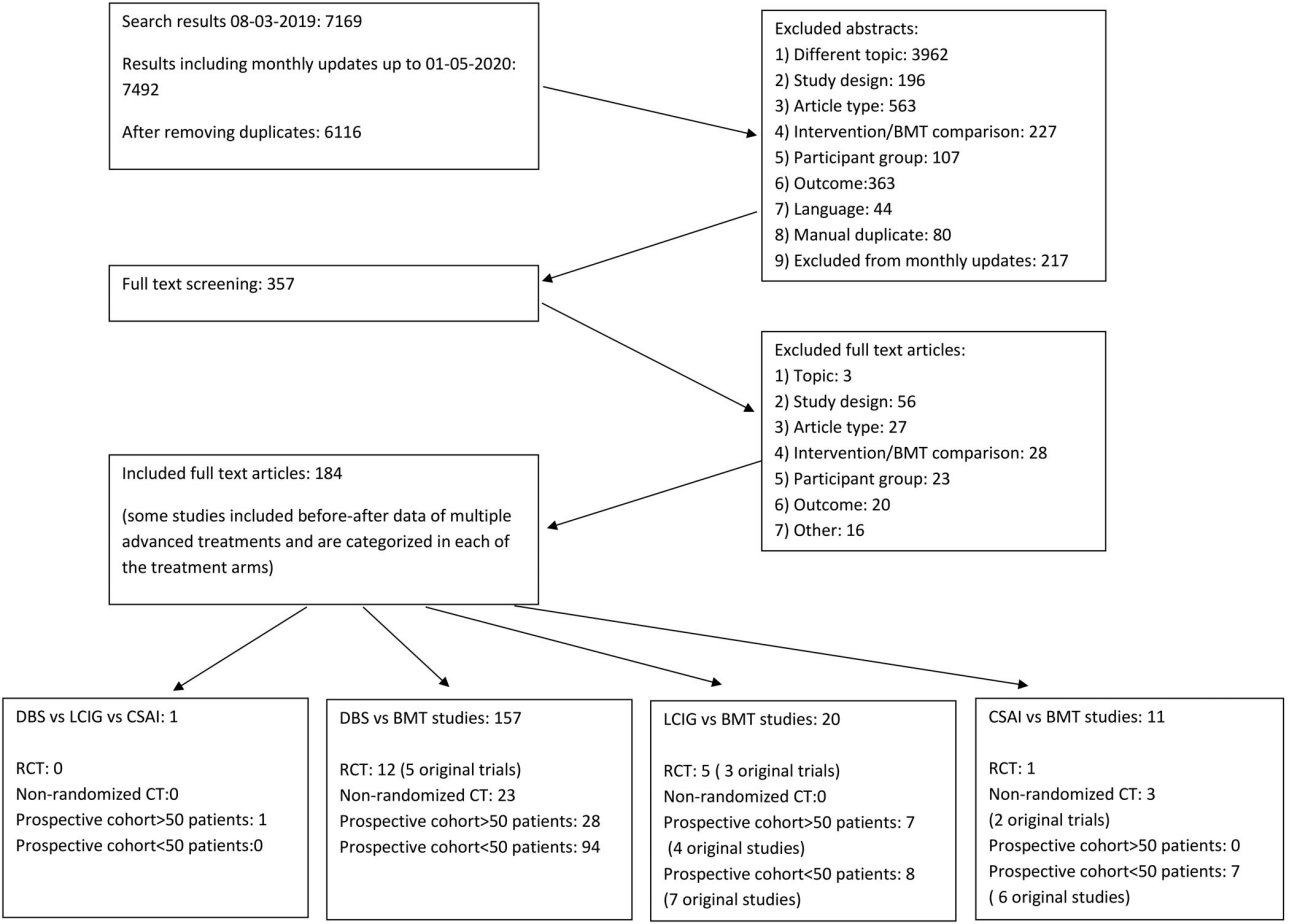
Flowchart of search and study results. BMT, best medical treatment; CSAI, continuous subcutaneous apomorphine infusion; DBS, beep brain stimulation; LCIG, levodopa‐carbidopa intestinal gel; RCT, randomized controlled trial.

Figure 2 summarizes the results of the search and study selection. After we screened 6116 titles and abstracts, 357 articles were selected for full‐text review. Of these, 184 articles met inclusion criteria. Of these, 157 were DBS studies, of which many were either DBS non‐RCTs (n = 23) or DBS prospective cohort studies (n = 122) and were not included in the meta‐analysis. Studies included were conducted in the United States (US) (n = 3), Europe (n = 21), the Middle East (n = 1), Australia (n = 1), and on multiple continents (n = 4). One study compared all three advanced treatments in a comparative, prospective cohort study.^18^ For DBS, we retrieved 12 articles from five original RCTs.^19^, ^20^, ^21^, ^22^, ^23^, ^24^, ^25^, ^26^, ^27^, ^28^, ^29^, ^30^ They all had moderate to high levels of evidence for all outcomes, so we excluded other study designs. For LCIG, five articles based on three unique RCTs fulfilled criteria for further analysis.^31^, ^32^, ^33^, ^34^, ^35^ We also included 16 prospective cohort studies,^36^, ^37^, ^38^, ^39^, ^40^, ^41^, ^42^, ^43^, ^44^, ^45^, ^46^, ^47^, ^48^, ^49^, ^50^, ^51^ as some outcomes of interest only reached a low certainty of evidence. For CSAI we included one RCT comparing CSAI with BMT^52^ and two non‐RCTs (three articles).^53^, ^54^, ^55^ As these studies did not have high‐quality evidence for all outcomes, six prospective cohorts (seven articles) were also included.^36^, ^56^, ^57^, ^58^, ^59^, ^60^, ^61^ Table 1 describes the characteristics of the studies included in the GRADE analysis.

### 2.2 Population Characteristics

Sample sizes ranged from 10 to 366 participants. The total studies in the meta‐analysis included 1048 patients in the DBS group, 106 in the LCIG group (1066 when including prospective cohorts), and 107 in the CSAI group (286 when including prospective cohorts). The definition of advanced PD in most studies was not specified other than motor fluctuations and/or dyskinesia despite BMT. Some used key indicators such as at least 3 hours of OFF time per day, at least 3–5 years of PD, and BMT. The mean age of participants in the CSAI studies ranged from 56 to 65 years. The mean age in the DBS trials ranged from 59 to 62 years, apart from the two studies that included substantially younger patients (mean age 48–53 years).^20^, ^21^ These studies also had patients with a shorter duration of disease compared to the other DBS studies, but all included patients with motor complications and were all included in the meta‐analysis. The LCIG population had a wider age range (52–74 years). Three studies included predominantly women,^40^, ^46^, ^58^ in all other studies the number of men dominated, or there was an equal distribution. Disease severity was measured differently across studies and was therefore difficult to compare. Disease duration was not available for all studies: three DBS RCTs reported mean disease durations of 6.4 to 11.5 years^20^, ^21^, ^22^; the two other reported duration of levodopa treatment of 10.8–13.6 years. The largest LCIG RCT^31^ reported a mean duration of 10–11.8 years, one RCT reported a median duration of 13 years,^32^ and the smallest did not report disease duration.^33^ The LCIG cohort studies reported mean durations of 10.5–16.1 years. The CSAI RCT population had a mean duration of 10.6–11.8 years.^52^ The other CSAI studies reported mean durations of 10.0–14.5 years. More details on population demographics of the included studies can be found in Supplement S1.

### 2.3 Intervention and Comparison Characteristics

All five DBS RCTs compared DBS combined with BMT versus BMT alone. In four studies, DBS targeted the subthalamic nucleus (STN),^19^, ^20^, ^21^, ^22^ or patients were equally randomized to subthalamic nucleus DBS or globus pallidus nucleus DBS.^23^ Three studies were of bilateral stimulation^19^, ^21^, ^23^; in one most patients had unilateral stimulation^20^; and one did not reveal whether participants received unilateral or bilateral stimulation.^22^

All LCIG studies used LCIG combined with BMT versus placebo infusion combined with BMT or LCIG combined with BMT versus BMT alone. Mean levodopa infusion dosage was only mentioned in one RCT (average 94 mg/h, range 26 to 196 mg/h), and infused daily levodopa doses ranged between 456 and 3556 mg.^32^ The other two RCTs^31^, ^33^ mentioned total levodopa equivalent doses, which included other parkinsonian medications. In all three RCTs, continuous infusion was applied during waking hours (8–16 h daily). Seven cohort studies^36^, ^39^, ^40^, ^43^, ^44^, ^45^, ^48^ reported infused daily levodopa doses between 1127 and 1840 mg. In the cohort studies, LCIG was sporadically continued at night. Five LCIG cohort studies^40^, ^44^, ^45^, ^48^, ^51^ reported percentage of patients on LCIG monotherapy of 28% to 97%. Two LCIG RCTs used a nasoduodenal tube for LCIG and not a PEG‐J tube.^32^, ^33^ In the largest LCIG RCT (n = 71), controls also had PEG‐J tube placement but received placebo.^31^

All CSAI studies compared CSAI combined with BMT versus BMT alone. Although not all studies provided details, most studies used antiemetics to reduce the risk of side effects. Most studies had continuous infusion during waking hours, but only one continued CSAI at night.^57^ Mean apomorphine infusion dosage ranged from 73 to 113 mg/day. In the CSAI RCT, CSAI combined with BMT was compared to placebo infusion combined with BMT using the same procedures as in the CSAI group.^52^

### 2.4 Follow‐Up Characteristics

Follow‐up ranged from 4 days to 5 years. The DBS RCTs had longer follow‐up (6–24 months)^19^, ^20^, ^21^, ^22^, ^23^ than the other two advanced therapy RCTs.^31^, ^32^, ^33^, ^52^ LCIG RCTs follow‐up ranged from 4 days to 12 weeks.^31^, ^32^, ^33^ In the non‐randomized LCIG studies, follow‐up ranged from 6 months to 4 years.^36^, ^37^, ^38^, ^39^, ^40^, ^41^, ^42^, ^43^, ^44^, ^45^, ^46^, ^47^, ^48^, ^49^, ^50^, ^51^ The CSAI RCT had a follow‐up of 12 weeks.^52^ In non‐randomized CSAI studies, follow‐up ranged from 6 months to 2 years.^36^, ^44^, ^45^, ^56^, ^57^, ^58^, ^59^, ^60^, ^61^

### 2.5 Classification of Evidence

Table 2 shows the MCID for each outcome and the certainty of evidence per outcome, and Tables S3–S5 describe the classification of evidence using GRADE for each treatment. The quality and certainty of evidence for QoL was high for DBS. For the other outcomes, quality of evidence for DBS was downgraded to moderate for inconsistency (Table S3). The quality of evidence of LCIG RCTs was moderate for QoL, ADL, and ON and OFF time. Even though there were three RCTs, no meta‐analyses were conducted due to the study design of the two crossover trials (details in Table S4). Main reasons for downgrading were inconsistency and imprecision (Table S4). The quality of evidence of the LCIG cohort studies was very low for all outcomes. For ON and OFF time and serious adverse events (SAEs), the CSAI RCT had a moderate level of certainty. For QoL, the study was downgraded for imprecision, as the confidence intervals included MCID and scored a low level of certainty (Table S5). The details of the GRADE judgements are available upon request.

**TABLE 2:** Outcomes and clinically relevant effects

*Quality of Life (PDQ‐39/PDQ‐8) (MCID PDQ‐39 = −4.72, MCID PDQ‐8 = −5.94)*								
Treatment	Absolute effect mean treatment difference (CI)	Participants (n) (studies)	Certainty of evidence (GRADE)	MD AT before−after (SD)	MD BMT before−after (SD)	Clinically relevant effect intervention	Clinical effect BMT	Difference AT vs. BMT
DBS vs. BMT	−7.4 (−9.3; −5.6)	980 (5 RCTs)	High	−7.2 (13.45)	0.2 (10.75)	If you treat 100 patients with DBS, **57** will have a clinically significant effect	If you treat 100 patients with BMT, **32** will have a clinically significant effect	**25** more patients in the DBS group will experience a beneficial effect
LCIG vs. BMT	−7.0 (−16.0; 2.0)	66 (1 RCT)	Moderate	−10.9 (19.5)	−3.9 (17.8)	If you treat 100 patients with LCIG, **62** will have a clinically significant effect	If you treat 100 patients with BMT, **48** will have a clinically significant effect	**14** more patients in the LCIG group will experience a beneficial effect
CSAI vs. BMT	−2.5 (−7.6; 2.7)	106 (1 RCT)	Low	−0.1 (14.37)	2.4 (11.83)	If you treat 100 patients with CSAI, **34** will have a clinically significant effect	If you treat 100 patients with BMT, **24** will have a clinically significant effect	**10** more patients in the CSAI group will experience a beneficial effect

*Activities of daily living (UPDRS II) (MCID =* −*3)*								
Treatment	Absolute effect mean treatment difference (CI)	Number of participants (number of studies)	Certainty of evidence (GRADE)	MD AT before−after (CI)	MD BMT before−after (CI)	Clinically relevant effect intervention	Clinical effect BMT	Difference AT vs. BMT
DBS vs. BMT	−1.9 (−3.8; −0.0)	949 (5 RCTs)	Moderate	−0.9 (5.98)	0.7 (4.56)	If you treat 100 patients with DBS, **36** will have a clinically significant effect	If you treat 100 patients with BMT, **21** will have a clinically significant effect	**15** more patients in the DBS group will experience a beneficial effect
LCIG vs. BMT	−3.0 (−5.3; − 0.8)	66 (1 RCT)	Moderate	−1.8 (7.7)	1.3 (7.7)	If you treat 100 patients with LCIG, **44** will have a clinically significant effect	If you treat 100 patients with BMT, **29** will have a clinically significant effect	**15** more patients in the LCIG group will experience a beneficial effect
CSAI vs. BMT	NA	(3 observational studies)	NA	NA	NA	The studies used different scales and therefore an estimate could not be given	The studies used different scales and therefore an estimate could not be given	The studies used different scales and therefore an estimate could not be given

*ON time (MCID = 1)*								
Treatment	Absolute effect mean treatment difference (CI)	Number of participants (number of studies)	Certainty of evidence (GRADE)	MD AT before‐ after (SD)	MD BMT before‐after (SD)	Clinically relevant effect intervention	Clinical effect BMT	Difference AT vs. BMT
DBS vs. BMT	3.8 (2.0; 5.6)	584 (3 RCTs)	Moderate	3.7 (4.61)	−0.0 (4.12)	If you treat 100 patients with DBS, **72** will have a clinically significant effect	If you treat 100 patients with BMT, **40** will have a clinically significant effect	**32** more patients in the DBS group will experience a beneficial effect
LCIG vs. BMT	1.9 (0.6; 3.2)	66 (1 RCT)	Moderate	4.1 (4.43)	2.2 (4.23)	If you treat 100 patients with LCIG, **76** will have a clinically significant effect	If you treat 100 patients with BMT, **62** will have a clinically significant effect	**14** more patients in the LCIG group will experience a beneficial effect
CSAI vs. BMT	2.0 (0.7; 3.2)	106 (1 RCT)	Moderate	2.8 (3.26)	0.8 (2.93)	If you treat 100 patients with CSAI, **71** will have a clinically significant effect	If you treat 100 patients with BMT, **47** will have a clinically significant effect	**24** more patients in the CSAI group will experience a beneficial effect

*OFF time (MCID =* −*1)*								
Treatment	Absolute effect mean treatment difference (CI)	Number of participants (number of studies)	Certainty of evidence (GRADE)	MD AT before‐ after (SD)	MD BMT before‐after (SD)	Clinically relevant effect intervention	Clinical effect BMT	Difference AT vs. BMT
DBS vs. BMT	−2.8 (−4.2; −1.4)	585 (3 RCTs)	Moderate	−2.81 (4.31)	−0.0 (5.01)	If you treat 100 patients with DBS, **66** will have a clinically significant effect	If you treat 100 patients with BMT, **42** will have a clinically significant effect	**24** more patients in the DBS group will experience a beneficial effect
LCIG vs. BMT	−1·9 (−3·1; −0·8)	66 (1 RCT)	Moderate	−4.04 (3.85)	−2.1 (3.67)	If you treat 100 patients with LCIG, **79** will have a clinically significant effect	If you treat 100 patients with BMT, **62** will have a clinically significant effect	**17** more patients in the LCIG group will experience a beneficial effect
CSAI vs. BMT	−1.9 (−3.2; −0.6)	106 (1 RCT)	Moderate	−2.47 (3.7)	−0.6 (2.80)	If you treat 100 patients with CSAI, **65** will have a clinically significant effect	If you treat 100 patients with BMT, **44** will have a clinically significant effect	**21** more patients in the CSAI group will experience a beneficial effect

*Serious adverse events*								
Treatment	Relative risk (CI)	Number of participants (number of studies)	Certainty of evidence (GRADE)	Proportion of participants with event (CI) Intervention	Proportion of participants with event (CI) BMT	Serious adverse events intervention	Serious adverse events BMT	Difference AT vs. BMT
DBS vs. BMT	2.31 (1.25; 4.28)	907 (4 RCTs)	Moderate	0.34 (0.20; 0.52)	0.14 (0.06; 0.32)	If you treat 100 patients with DBS, **34** will have a serious adverse event	If you treat 100 patients with BMT, **14** will have a serious adverse event	**20** more patients in the DBS group will experience a serious adverse event
LCIG vs. BMT	0.66 (0.23; 1.87)	71 (1 RCT)	Low	0.14 (0.05; 0.29)	0.21 (0.09; 0.38)	If you treat 100 patients with LCIG, **14** will have a serious adverse event	If you treat 100 patients with BMT, **21**will have a serious adverse event	**7** fewer patients in the LCIG group will experience a serious adverse event
CSAI vs. BMT	2.45 (0.5; 12.1)	106 (1 RCT)	Moderate	0.09 (0.03; 0.2)	0.04 (0.0; 0.13)	If you treat 100 patients with CSAI, **9** will have a serious adverse event	If you treat 100 patients with BMT, **4** will have a serious adverse event	**5** more patients in the CSAI group will experience a serious adverse event

Serious adverse events: events leading to new or prolonged hospital admission with death, disability, or serious health impairment.

Abbreviations: PDQ, Parkinson's Disease Questionnaire; MCID, minimal clinical important difference; CI, confidence interval; GRADE, Grading of Recommendations, Assessment, Development, and Evaluation; MD, mean difference; AT, advanced therapy; SD, standard deviation; BMT, best medical treatment; DBS, deep brain stimulation; RCT, randomized controlled trial; LCIG, levodopa‐carbidopa intestinal gel; CSAI, continuous subcutaneous apomorphine infusion; UPDRS II, Unified Parkinson's Disease Rating Scale Part II; NA, not available.

### 2.6 Effectivity

#### 2.6.1 Quality of Life

For QoL, DBS had the largest clinically relevant effect, as 25 more patients had improved QoL compared to BMT when 100 patients were treated. The LCIG RCTs were not pooled, as the randomized crossover trial^32^ measured treatment difference between LCIG and BMT but did not report change from baseline. The RCT of LCIG versus BMT^31^ had a similar mean treatment difference (−7.0 points) as DBS versus BMT (−7.4 points). However, the 95% confidence intervals (95% CIs) were much wider for LCIG versus BMT (95% CI −16.0; 2.0) compared with DBS versus BMT (95% CI −9.3; −5.6). This produced a smaller chance of beneficial effect of 14 more patients improving in QoL when treated with LCIG compared to BMT for 100 patients treated. The BMT group in the LCIG RCT^31^ also experienced a beneficial effect on QoL (−3.9 points). The cohort studies showed a larger effect,^36^, ^38^, ^39^, ^40^, ^41^, ^43^, ^44^, ^45^, ^46^, ^47^, ^48^ but did not have a BMT comparison group and had a lower level of certainty due to risk of bias, inconsistency, and imprecision. There was a small beneficial effect of CSAI in the RCT, with 10 more patients (out of 100) improving with CSAI compared to BMT. The non‐RCT^55^ (−32.2 points on PDQ‐39 [95% CI −47.7; −16.6]) and before−after study (−14.8 points [95% CI −20.0; −9.6])^36^ showed much larger effects on QoL, with a lower level of certainty due to risk of bias and imprecision. The non‐RCT also had significant differences in QoL at baseline between the CSAI group and the BMT group.^55^

#### 2.6.2 Activities of Daily Living

DBS versus BMT had a pooled treatment effect of −1.9 points (95% CI −3.8; 0.0) on the UPDRS II, and LCIG versus BMT had a mean treatment effect of −3.0 points (95% CI −5.3; −0.8). Taking the mean differences of each treatment, the CIs and the MCID for ADL (−3), DBS, and LCIG had similar effects, with 15 more patients having a clinically relevant beneficial effect compared with BMT for 100 treated patients. The LCIG RCTs were not pooled, as the randomized crossover trial^32^ measured treatment difference between LCIG and BMT and did not report change from baseline. The LCIG cohort studies with follow‐up ≤1 year showed similar effects (−4.0 points [95% CI −4.6; −3.4]),^38^, ^42^, ^43^, ^46^, ^47^, ^48^ and the cohort studies with follow‐up of 2 to 3 years showed no effect (0.3 points [95% CI −2.6; 3.1]).^38^, ^40^, ^44^, ^45^ The CSAI RCT^52^ did not use ADL as an outcome measure. The cohort studies used different rating scales and different methods to report the effects (percentage improvement, absolute difference, percentage of patients improved) and could not be pooled.

#### 2.6.3 ON and OFF Time Duration

With CSAI, 21 more patients had a clinically relevant beneficial effect on OFF time duration compared with BMT for 100 treated patients. A similar effect was seen for ON time duration, as 24 more patients in the CSAI group had a beneficial effect compared with BMT. DBS had a similar effect on OFF time duration as CSAI, with 24 more patients having a beneficial effect compared to CSAI. Some 32 more patients in the DBS group compared with the BMT group had a beneficial effect on ON time duration. For LCIG, the small beneficial effect in reduction in OFF time duration was also reflected in a small beneficial effect in increased ON time due to a similar large effect in the LCIG group as BMT in the LCIG RCT.^31^ The LCIG RCTs were not pooled, as the randomized crossover trial^33^ measured treatment difference between LCIG and BMT and did not report change from baseline.

#### 2.6.4 Serious Adverse Effects

The risk for an SAE is highest for DBS, with a chance of 20 more patients experiencing an SAE compared to BMT. In two RCTs,^22^, ^23^ the majority of SAEs were surgery‐, device‐, or stimulation‐related. In the LCIG group, two RCTS used nasoduodenal tubes instead of a PEG ‐J tube, which excluded surgical risks. The SAEs are given for the RCT using a PEG‐J tube. The risk of an SAE in the LCIG group was less than in the BMT group, with six fewer patients experiencing one. The adverse events were almost all due to surgery and device‐related complications in both the LCIG and BMT group, who also underwent the PEG‐J tube placement.^31^ The risk was small for CSAI treatment, with a chance of five more patients in the CSAI group experiencing an SAE compared to BMT. The SAEs reported in the CSAI RCT were all considered treatment‐related.^52^

## 3 Discussion

In this systematic review and meta‐analysis, we compared the effects and risks of CSAI, DBS, and LCIG for outcomes that were patient‐selected. There is increasing awareness that patient involvement in decision‐making is important for several reasons, of which the greatest is the ethical imperative.^62^ Furthermore, studies suggest that shared decision‐making (SDM) improves patient satisfaction and treatment adherence.^63^ In order to be really involved in decision‐making, patients should have access to information that is relevant to them.^64^ Although outcomes were not determined by patients in previous reviews,^5^, ^6^ the patients in our study selected almost similar outcomes. Interestingly, when we asked seven Dutch movement disorder specialists to prioritize the most relevant outcome measures concerning advanced treatment, they agreed with the patients' outcomes but wanted to include the UPDRS III. We conducted the meta‐analysis for this outcome for the neurologists but did not include the results in this review, because we focused on the patient perspective. Caregiver perspective is also important to consider in the overall decision process, in particular in people with advanced PD due to cognitive decline who may find it difficult to participate in the decision process. That is one of the reasons we included caregivers in the focus groups, which was the actual starting point for the development process. The changing role for the partner linked to a specific advanced treatment emerged as a factor in treatment choice in the focus groups.^8^ In the subsequent questionnaire, however, this factor was given the lowest priority, and we did not select caregiver burden as an outcome for the evidence synthesis.^4^ Importantly, we added an extra step in this review, namely to convert the evidence to patient‐tailored and comprehensible information. Until now, patients (and neurologists) lacked appropriate information to benchmark the various advanced treatments.^8^ We found no RCTs that directly compared all three therapies. We found evidence for each advanced therapy compared to BMT for most outcomes. In traditional systematic reviews and meta‐analyses, interpretation of the results in clinical practice is difficult. Specifically, the relevance of a statistically significant effect is limited in clinical practice,^65^ and interpretation of the weighted mean difference often depends on what the decision‐maker considers a relevant estimate of a specific outcome effect. This data interpretation can even differ among experienced meta‐analysis reviewers.^66^ To make the interpretation clinically more relevant, the MCID is an important first step.^67^ MCIDs were available in the literature for all outcomes selected by patients. To make the best available evidence comparable for patients, we used the cumulative distribution function and the MCID. With this approach, the evidence can be compared for the different treatments relative to BMT.

Discussing the evidence with a patient in the decision process should not be limited to the effects and adverse events of the treatment options, but should also include the level of certainty about the effect. After previous reviews were published, the most important updates included new RCTs for CSAI and LCIG, increasing the quality of evidence for these treatments. The level of certainty of the evidence was highest for DBS versus BMT. Also, DBS had the largest beneficial effects on QoL, ADL, and ON time, and the highest risk for an SAE. LCIG showed smaller beneficial effects than DBS on QoL, ON time, and OFF time. The total levodopa dosage increased in the BMT group during the trial in the most recent LCIG RCT, which could imply that they had not received BMT yet at the start of the study.^31^ This would also explain the relatively large beneficial effect in the BMT group and therefore the lower mean treatment difference effect between LCIG and BMT. Another explanation could be that there was a larger placebo effect in the BMT group, as the patients in the BMT group received a PEG‐J tube as well, while the BMT group in the DBS studies did not receive sham surgery. The original authors stated that LCIG has similar effects on QoL and ON and OFF time as DBS, but in our meta‐analysis the effects of DBS are higher. The mean treatment difference for QoL in DBS versus BMT is similar to LCIG versus BMT; however, due to the broader confidence intervals in the LCIG trial compared to the pooled DBS trials, the probability of a clinically relevant effect (based on the MCID) is lower. This illustrates how our approach also includes the level of certainty of an effect (the smaller the confidence intervals, the larger the clinically relevant effect); this information is helpful for decision‐making, as it shows that the certainty about the evidence for DBS is greater than for the other two advanced treatments. The lower risk of SAEs for LCIG compared to DBS should be interpreted cautiously, as the BMT group in the LCIG RCT also received PEG‐J tube placement, and most adverse events were device‐ or surgery‐related. CSAI had smaller beneficial effects on QoL than DBS or LCIG. CSAI had the lowest risk of an SAE. The differences in certainty of evidence can be discussed with the patient. The calculation based on the cumulative distribution function helps the patient to have a more realistic expectation of the effect of an advanced therapy, as the calculation includes the imprecision of the estimate. Literature on communicating evidence to patients is largely based on how to effectively translate population‐based evidence to the individual patient, showing most communication tools (either verbal, written, or computer‐based) will increase patient understanding, but will increase understanding more if the tool is tailored, structured, or interactive, such as decision aids.^68^ The best method to discuss the level of evidence to increase interpretation is less well evaluated, but suggestions are to increase understanding by using common symbols and words (provided in GRADE software).^69^

Incorporating these findings into patient information will better equip them to make an informed decision. The patient‐relevant outcomes for the three advanced treatments compared to BMT can be presented on a single page in comprehensible numerical information. This can serve as a catalyst for shared decision‐making. Effects and risks are better understood when displayed in simple frequencies (x out of 100 patients develop…) and presented in absolute risks instead of relative risks. Graphs can help patients to better understand incremental effect/risk formats (absolute effect/risk increase or decrease).^70^ This review shows that all therapies have beneficial effects, with DBS having the highest level of evidence. Previous reviews showed similar results, but they did not have sufficient data on QoL.^5^, ^6^ The National Institute for Health and Care Excellence (NICE) guideline recommends DBS for advanced PD patients, but describes apomorphine infusion therapy as one of the treatments under BMT, which makes it difficult to compare their recommendation to our findings. LCIG is not recommended by NICE, as it was deemed not to be cost effective in daily practice.^71^ However, it is possible to recommend LCIG to a patient, because personalized treatment advice is based on many factors which extend beyond the strict evidence derived from evidence‐based guidelines. The first step is to determine the nature and severity of advanced PD symptoms, their responsiveness to an adequate trial of levodopa, and to assess the presence of comorbidity and cognitive and neuropsychiatric status. Second, it is necessary to decide about the presence of absolute and relative contraindications for any of the advanced treatments. Third, an individual risk–benefit assessment must be made based on individual patient preferences as to which treatment would be of greatest benefit.^6^ This information about treatments should be tailored to the patient's main goal of treatment, the risks the patient is willing to accept, the social support system, and practical therapy preferences (daily care, number of follow‐ups needed, and the likely influence on social life).^6^ This requires an individually tailored decision process and will not necessarily lead to a choice for the treatment with the largest effect. These results provide patients, caregivers, and healthcare professionals in the field with a useful and comprehensive tool in the shared decision process.

### 3.1 Strengths and Limitations

The main strength of this study is that we conducted a systematic review from the patient perspective. Furthermore, we included the certainty of evidence and converted the results into information relevant to the patient. This makes it applicable for clinical dialogue between patient, caregiver, and clinician in the decision‐making process. This review is not without shortcomings. A large limitation is the lack of direct comparative studies including all three therapies. Furthermore, the level of certainty of evidence is different for different treatments. Including all studies resulted in more heterogenous groups and could have influenced the effects. One DBS study, for example, included unilateral stimulation, which is no longer common practice,^20^ and the DBS studies had longer follow‐up compared to the CSAI and LCIG RCTs. Longer follow‐up increases the risk of progression of PD and decreases the measured treatment effects. In general, the follow‐up durations of the RCTs were limited (up to 3 months for LCIG and CSAI, and up to 24 months for DBS). PD patients indicated that they preferred to have more information on the long‐term effects, but did not rank this as most important in reaching the decision for one of the advanced treatments.^4^ For DBS, improvements have been shown for follow‐ups as long as 10 years, but these studies often had several limitations, such as bias due to losses to follow‐up and deterioration due to natural disease progression.^72^ In the LCIG cohort studies, QoL improvement remained, but ADL improvement disappeared after a follow‐up of up to 2 to 3 years. More importantly, these LCIG studies were characterized by loss to follow‐up or discontinuation of treatment. For CSAI, the long‐term effects were mainly derived from retrospective studies, which showed prolonged benefits, but with the same limitation of a substantial number of patient withdrawals.^73^

Further high‐quality studies are needed for LCIG and CSAI and should include QoL. This is a key outcome measure, because patients themselves ranked this as the most important outcome, and also because improvements in ON or OFF time (helpful as it may be as a useful intermediate outcome) may not necessarily translate into a better QoL. A further important reason for including QoL as a critical outcome is that patients who undergo a device‐aided therapy will experience residual non‐motor symptoms, some of which will not improve after starting the treatment, even when it is very effective in reducing the level of motor disability and alleviating motor fluctuations. Including QoL as an outcome will capture the overall status of the patient following the intervention, including the non‐motor burden that may accumulate due to natural disease progression. From a patient perspective, upfront information about these residual non‐motor symptoms might very well be an important factor in choosing a specific treatment.

Another limitation is the difference in populations, though all patients in all studies were defined as patients with advanced PD with motor fluctuations despite BMT, disease durations in the DBS RCTs were shorter, and data suggest that not all populations were already optimally treated with oral medication. It is often difficult to extrapolate the findings from trials to real life due to inclusion of often non‐representative populations. For example, in daily practice we often see elderly patients with response fluctuations and with relevant comorbidity, but they are usually excluded from participation in trials. The next level of decision‐making would be to create more personalized predictions of both the effects and risks using big data analytics of large naturalistic and typically more heterogeneous patient populations, based on individual characteristics such as age, comorbidity, or cardinal symptom.^3^ We strongly recommend that the patient's voice should be included in future clinical trials from inception to design (including the primary outcome choice) and the ultimate communication in a publication. Consistently including the patients' voices might also help reduce the heterogeneity of outcome measures across different studies, thereby facilitating their interpretation. However, a lack of studies comparing the three treatments does not mean there is no information to compare them. Using the GRADE approach to present the level of certainty about the evidence and the MCID and cumulative distribution function to present the effects and risks can support both the patient (and presumably the clinician) in balancing the benefits and risks for each treatment option. This method can serve as a template for translating evidence into patient‐relevant information for many other decisions and has the benefit of looking beyond statistical significance to clinical relevance that is meaningful to patients. Our meta‐analysis is informed by studies where the measurement was based on a snapshot clinical judgement by an observer, typically using a subjectively rated clinical scale (which is hampered by their subjective nature, as well as by the natural day‐to‐day fluctuations in patient performance), or based on (retrospective) patient reports (which are hampered by poor patient recall). Future studies would greatly benefit from having objective and longitudinal measurements in a patient's own living circumstances, for example, using body‐worn sensors.^74^, ^75^ The choice for cut‐off levels of MCIDs is debatable. All MCIDs were determined using the anchor‐based method or a combination of methods including at least the anchor‐based method.^13^, ^14^, ^15^ The strength of anchor‐based methods is that the MCIDs are determined by patient assessments and not expert opinion (consensus methods) or statistics driven (distribution methods).^67^ However, they also carry a potential risk of bias (subjectively chosen anchor, recall bias regarding change of symptoms).^15^, ^67^

## 4 Conclusions

In this systematic review and meta‐analysis, we compared the effects and risks of DBS, LCIG, and CSAI for patient‐selected outcomes, considering the level of certainty about the evidence. The evidence was transformed into comprehensible and comparable information that is easy to read and understand by patients. These data are of great value in daily clinical dialogue between patient, caregiver, and clinician in the decision‐making process for an advanced treatment in PD.

## Author Roles

(1) Research Project: A. Conception, B. Organization, C. Execution; (2) Statistical Analysis: A. Design, B. Execution, C. Review and Critique; (3) Manuscript Preparation: A. Writing of the First Draft, B. Review and Critique.

F.A.P.N.: 1A, 1B, 1C, 2A, 2B, 2C, 3A, 3B

R.E.: 2C, 3B

R.M.A.B: 3B

H.G.: 2A, 2B

B.R.B: 1A, 2A, 2C, 3B

B.P.: 1A, 1B, 1C, 2A, 2C, 3A, 3B

M.J.M.: 1A, 1B, 2A, 2C, 3A, 3B

## Full Financial Disclosures for the Previous 12 Months

The Radboud University Medical Center received unrestricted grants from Medtronic, Abbvie, and Apotheekzorg for developing a decision aid for advanced therapies in Parkinson's disease. F.A.P.N., B.P., M.J.M., and B.R.B. participated in this study.

F.A.P.N. reports no other conflicts of interest. R.E. reports no conflicts of interest. R.M.A.B. reports no conflicts of interest. H.G. reports no conflicts of interest. B.R.B. currently serves as Editor‐in‐Chief for the *Journal of Parkinson's Disease*, serves on the editorial board of *Practical Neurology* and *Digital Biomarkers*, has received honoraria for serving on the scientific advisory board for Zambon, Biogen, UCB, and Walk with Path, has received fees for speaking at conferences from AbbVie, Zambon, Roche, GE Healthcare, and Bial, and has received research support from the Netherlands Organization for Scientific Research, the Michael J. Fox Foundation, UCB, Abbvie, Zambon, the Stichting Parkinson Fonds, the Hersenstichting Nederland, the Parkinson's Foundation, Verily Life Sciences, Horizon 2020, the Topsector Life Sciences and Health, and the Parkinson Vereniging. B.P. reports no other conflicts of interest. M.J.M. reports no other conflicts of interest.

## Supporting information


**Appendix S1**. Supplementary Information

## References

[1] Chapuis S , Ouchchane L , Metz O , Gerbaud L , Durif F . Impact of the motor complications of Parkinson's disease on the quality of life. Mov Disord 2005;20:224–230. 15384126 10.1002/mds.20279

[2] Sackett DL , Rosenberg WM , Gray JA , Haynes RB , Richardson WS . Evidence based medicine: what it is and what it isn't. 1996. Clin Orthop Relat Res 2007;455:3–5. 17340682

[3] van den Heuvel L , Dorsey RR , Prainsack B , et al. Quadruple decision making for Parkinson's disease patients: combining expert opinion, patient preferences, scientific evidence, and big data approaches to reach precision medicine. J Parkinsons Dis 2020;10:223–231. 31561387 10.3233/JPD-191712 PMC7029360

[4] Nijhuis FAP , van den Heuvel L , Bloem BR , Post B , Meinders MJ . The patient's perspective on shared decision‐making in advanced Parkinson's disease: a cross‐sectional survey study. Front Neurol 2019;10:896. 31474936 10.3389/fneur.2019.00896 PMC6706819

[5] Clarke CE , Worth P , Grosset D , Stewart D . Systematic review of apomorphine infusion, levodopa infusion and deep brain stimulation in advanced Parkinson's disease. Parkinsonism Relat Disord 2009;15:728–741. 19805000 10.1016/j.parkreldis.2009.09.005

[6] Volkmann J , Albanese A , Antonini A , et al. Selecting deep brain stimulation or infusion therapies in advanced Parkinson's disease: an evidence‐based review. J Neurol 2013;260:2701–2714. 23287972 10.1007/s00415-012-6798-6 PMC3825542

[7] Edwards A , Elwyn G . Understanding risk and lessons for clinical risk communication about treatment preferences. Qual Health Care 2001;10(Suppl 1):i9–i13. 11533431 10.1136/qhc.0100009.. PMC1765742

[8] Nijhuis FA , van Heek J , Bloem BR , Post B , Faber MJ . Choosing an advanced therapy in Parkinson's disease; is it an evidence‐based decision in current practice? J Parkinsons Dis 2016;6:533–543. 27472888 10.3233/JPD-160816

[9] Antonini A , Stoessl AJ , Kleinman LS , et al. Developing consensus among movement disorder specialists on clinical indicators for identification and management of advanced Parkinson's disease: a multi‐country Delphi‐panel approach. Curr Med Res Opin 2018;34:2063–2073. 30016901 10.1080/03007995.2018.1502165

[10] Atkins D , Best D , Briss PA , et al. Grading quality of evidence and strength of recommendations. BMJ 2004;328:1490. 15205295 10.1136/bmj.328.7454.1490 PMC428525

[11] Higgins JP , Thompson SG . Quantifying heterogeneity in a meta‐analysis. Stat Med 2002;21:1539–1558. 12111919 10.1002/sim.1186

[12] Higgins JP , Thompson SG , Deeks JJ , Altman DG . Measuring inconsistency in meta‐analyses. BMJ 2003;327:557–560. 12958120 10.1136/bmj.327.7414.557 PMC192859

[13] Schrag A , Sampaio C , Counsell N , Poewe W . Minimal clinically important change on the unified Parkinson's disease rating scale. Mov Disord 2006;21:1200–1207. 16673410 10.1002/mds.20914

[14] Hauser RA , Auinger P , Parkinson Study Group . Determination of minimal clinically important change in early and advanced Parkinson's disease. Mov Disord 2011;26:813–818. 21437987 10.1002/mds.23638

[15] Horvath K , Aschermann Z , Kovacs M , et al. Changes in quality of life in Parkinson's disease: how large must they be to be relevant? Neuroepidemiology 2017;48:1–8. 28161701 10.1159/000455863

[16] Park KI . Fundamentals of Probability and Stochastic Processes with Applications to Communications. 1st ed. New York: Springer; 2018.

[17] Moher D , Liberati A , Tetzlaff J , Altman DG , PRISMA Group . Preferred reporting items for systematic reviews and meta‐analyses: the PRISMA statement. BMJ 2009;339:b2535. 19622551 10.1136/bmj.b2535 PMC2714657

[18] Dafsari HS , Martinez‐Martin P , Rizos A , et al. EuroInf 2: subthalamic stimulation, apomorphine, and levodopa infusion in Parkinson's disease. Mov Disord 2019;34:353–365. 30719763 10.1002/mds.27626

[19] Deuschl G , Schade‐Brittinger C , Krack P , et al. A randomized trial of deep‐brain stimulation for Parkinson's disease. N Engl J Med 2006;355:896–908. 16943402 10.1056/NEJMoa060281

[20] Schüpbach WM , Maltête D , Houeto JL , et al. Neurosurgery at an earlier stage of Parkinson disease: a randomized, controlled trial. Neurology 2007;68:267–271. 17151341 10.1212/01.wnl.0000250253.03919.fb

[21] Schuepbach WM , Rau J , Knudsen K , et al. Neurostimulation for Parkinson's disease with early motor complications. N Engl J Med 2013;368:610–622. 23406026 10.1056/NEJMoa1205158

[22] Williams A , Gill S , Varma T , et al. Deep brain stimulation plus best medical therapy versus best medical therapy alone for advanced Parkinson's disease (PD SURG trial): a randomised, open‐label trial. Lancet Neurol 2010;9:581–591. 20434403 10.1016/S1474-4422(10)70093-4 PMC2874872

[23] Weaver FM , Follett K , Stern M , et al. Bilateral deep brain stimulation vs best medical therapy for patients with advanced Parkinson disease: a randomized controlled trial. JAMA 2009;301:63–73. 19126811 10.1001/jama.2008.929 PMC2814800

[24] Daniels C , Krack P , Volkmann J , et al. Is improvement in the quality of life after subthalamic nucleus stimulation in Parkinson's disease predictable? Mov Disord 2011;26:2516–2521. 22170276 10.1002/mds.23907

[25] Witt K , Daniels C , Krack P , et al. Negative impact of borderline global cognitive scores on quality of life after subthalamic nucleus stimulation in Parkinson's disease. J Neurol Sci 2011;310:261–266. 21733529 10.1016/j.jns.2011.06.028

[26] Witt K , Daniels C , Reiff J , et al. Neuropsychological and psychiatric changes after deep brain stimulation for Parkinson's disease: a randomised, multicentre study. Lancet Neurol 2008;7:605–614. 18538636 10.1016/S1474-4422(08)70114-5

[27] Lhommee E , Wojtecki L , Czernecki V , et al. Behavioural outcomes of subthalamic stimulation and medical therapy versus medical therapy alone for Parkinson's disease with early motor complications (EARLYSTIM trial): secondary analysis of an open‐label randomised trial. Lancet Neurol 2018;17:211–212. 10.1016/S1474-4422(18)30035-8 29452685

[28] Rothlind JC , York MK , Carlson K , et al. Neuropsychological changes following deep brain stimulation surgery for Parkinson's disease: comparisons of treatment at pallidal and subthalamic targets versus best medical therapy. J Neurol Neurosurg Psychiatry 2015;86:622–629. 25185211 10.1136/jnnp-2014-308119

[29] Schuepbach WMM , Tonder L , Schnitzler A , et al. Quality of life predicts outcome of deep brain stimulation in early Parkinson disease. Neurology 2019;92:e1109–e1120. 30737338 10.1212/WNL.0000000000007037 PMC6442017

[30] Weaver FM , Follett KA , Stern M , et al. Randomized trial of deep brain stimulation for Parkinson disease: thirty‐six‐month outcomes. Neurology 2012;79:55–65. 22722632 10.1212/WNL.0b013e31825dcdc1 PMC3385495

[31] Olanow CW , Kieburtz K , Odin P , et al. Continuous intrajejunal infusion of levodopa‐carbidopa intestinal gel for patients with advanced Parkinson's disease: a randomised, controlled, double‐blind, double‐dummy study. Lancet Neurol 2014;13:141–149. 24361112 10.1016/S1474-4422(13)70293-X PMC4643396

[32] Nyholm D , Nilsson Remahl AI , Dizdar N , et al. Duodenal levodopa infusion monotherapy vs oral polypharmacy in advanced Parkinson disease. Neurology 2005;64:216–223. 15668416 10.1212/01.WNL.0000149637.70961.4C

[33] Kurth MC , Tetrud JW , Tanner CM , et al. Double‐blind, placebo‐controlled, crossover study of duodenal infusion of levodopa/carbidopa in Parkinson's disease patients with 'on‐off' fluctuations. Neurology 1993;43:1698–1703. 8414015 10.1212/wnl.43.9.1698

[34] Antonini A , Fung VS , Boyd JT , et al. Effect of levodopa‐carbidopa intestinal gel on dyskinesia in advanced Parkinson's disease patients. Mov Disord 2016;31:530–537. 26817533 10.1002/mds.26528 PMC5066747

[35] Isacson D , Bingefors K , Kristiansen IS , Nyholm D . Fluctuating functions related to quality of life in advanced Parkinson disease: effects of duodenal levodopa infusion. Acta Neurol Scand 2008;118:379–386. 18547273 10.1111/j.1600-0404.2008.01049.x

[36] Martinez‐Martin P , Reddy P , Katzenschlager R , et al. EuroInf: a multicenter comparative observational study of apomorphine and levodopa infusion in Parkinson's disease. Mov Disord 2015;30:510–516. 25382161 10.1002/mds.26067

[37] Valldeoriola F , Santacruz P , Rios J , et al. l‐Dopa/carbidopa intestinal gel and subthalamic nucleus stimulation: effects on cognition and behavior. Brain Behav 2017;7:e00848. 29201549 10.1002/brb3.848 PMC5698866

[38] Antonini A , Mancini F , Canesi M , et al. Duodenal levodopa infusion improves quality of life in advanced Parkinson's disease. Neurodegener Dis 2008;5:244–246. 18322402 10.1159/000113714

[39] Bohlega S , Abou Al‐Shaar H , Alkhairallah T , Al‐Ajlan F , Hasan N , Alkahtani K . Levodopa‐carbidopa intestinal gel infusion therapy in advanced Parkinson's disease: single middle eastern center experience. Eur Neurol 2015;74:227–236. 26618531 10.1159/000442151

[40] Caceres‐Redondo MT , Carrillo F , Lama MJ , et al. Long‐term levodopa/carbidopa intestinal gel in advanced Parkinson's disease. J Neurol 2014;261:561–569. 24477490 10.1007/s00415-013-7235-1

[41] Ciurleo R , Corallo F , Bonanno L , et al. Assessment of Duodopa® effects on quality of life of patients with advanced Parkinson's disease and their caregivers. J Neurol 2018;265:2005–2014. 29951701 10.1007/s00415-018-8951-3

[42] Palhagen SE , Dizdar N , Hauge T , et al. Interim analysis of long‐term intraduodenal levodopa infusion in advanced Parkinson disease. Acta Neurol Scand 2012;126:e29–e33. 22690905 10.1111/j.1600-0404.2012.01689.x

[43] Vijiaratnam N , Hewer S , Varley S , et al. Levodopa‐carbidopa intestinal gel: is the naso‐jejunal phase a redundant convention? Intern Med J 2018;48:469–471. 29623988 10.1111/imj.13754

[44] Zibetti M , Merola A , Ricchi V , et al. Long‐term duodenal levodopa infusion in Parkinson's disease: a 3‐year motor and cognitive follow‐up study. J Neurol 2013;260:105–114. 22772358 10.1007/s00415-012-6597-0

[45] Antonini A , Poewe W , Chaudhuri KR , et al. Levodopa‐carbidopa intestinal gel in advanced Parkinson's: final results of the GLORIA registry. Parkinsonism Relat Disord 2017;45:13–20. 29037498 10.1016/j.parkreldis.2017.09.018

[46] Antonini A , Yegin A , Preda C , Bergmann L , Poewe W . Global long‐term study on motor and non‐motor symptoms and safety of levodopa‐carbidopa intestinal gel in routine care of advanced Parkinson's disease patients; 12‐month interim outcomes. Parkinsonism Relat Disord 2015;21:231–235. 25585993 10.1016/j.parkreldis.2014.12.012

[47] Catalan MJ , Molina‐Arjona JA , Mir P , Cubo E , Arbelo JM , Martinez‐Martin P . Improvement of impulse control disorders associated with levodopa‐carbidopa intestinal gel treatment in advanced Parkinson's disease. J Neurol 2018;265:1279–1287. 29557989 10.1007/s00415-018-8803-1

[48] Fernandez HH , Standaert DG , Hauser RA , et al. Levodopa‐carbidopa intestinal gel in advanced Parkinson's disease: final 12‐month, open‐label results. Mov Disord 2015;30:500–509. 25545465 10.1002/mds.26123 PMC4674978

[49] Fernandez HH , Vanagunas A , Odin P , et al. Levodopa‐carbidopa intestinal gel in advanced Parkinson's disease open‐label study: interim results. Parkinsonism Relat Disord 2013;19:339–345. 23287001 10.1016/j.parkreldis.2012.11.020 PMC3661282

[50] Palhagen SE , Sydow O , Johansson A , et al. Levodopa‐carbidopa intestinal gel (LCIG) treatment in routine care of patients with advanced Parkinson's disease: an open‐label prospective observational study of effectiveness, tolerability and healthcare costs. Parkinsonism Relat Disord 2016;29:17–23. 27318707 10.1016/j.parkreldis.2016.06.002

[51] Slevin JT , Fernandez HH , Zadikoff C , et al. Long‐term safety and maintenance of efficacy of levodopa‐carbidopa intestinal gel: an open‐label extension of the double‐blind pivotal study in advanced Parkinson's disease patients. J Parkinsons Dis 2015;5:165–174. 25588353 10.3233/JPD-140456

[52] Katzenschlager R , Poewe W , Rascol O , et al. Apomorphine subcutaneous infusion in patients with Parkinson's disease with persistent motor fluctuations (TOLEDO): a multicentre, double‐blind, randomised, placebo‐controlled trial. Lancet Neurol 2018;17:749–759. 30055903 10.1016/S1474-4422(18)30239-4

[53] Di Rosa AE , Epifanio A , Antonini A , et al. Continuous apomorphine infusion and neuropsychiatric disorders: a controlled study in patients with advanced Parkinson's disease. Neurol Sci 2003;24:174–175. 14598073 10.1007/s10072-003-0116-0

[54] Morgante L , Basile G , Epifanio A , et al. Continuous apomorphine infusion (CAI) and neuropsychiatric disorders in patients with advanced Parkinson's disease: a follow‐up of two years. Arch Gerontol Geriatr Suppl 2004;9:291–296. 10.1016/j.archger.2004.04.039 15207426

[55] Martinez‐Martin P , Reddy P , Antonini A , et al. Chronic subcutaneous infusion therapy with apomorphine in advanced Parkinson's disease compared to conventional therapy: a real life study of non motor effect. J Parkinsons Dis 2011;1:197–203. 23934921 10.3233/JPD-2011-11037

[56] Stibe CM , Lees AJ , Kempster PA , Stern GM . Subcutaneous apomorphine in parkinsonian on‐off oscillations. Lancet 1988;1:403–406. 2893200 10.1016/s0140-6736(88)91193-2

[57] Pietz K , Hagell P , Odin P . Subcutaneous apomorphine in late stage Parkinson's disease: a long term follow up. J Neurol Neurosurg Psychiatry 1998;65:709–716. 9810943 10.1136/jnnp.65.5.709 PMC2170363

[58] Kanovsky P , Kubova D , Bares M , et al. Levodopa‐induced dyskinesias and continuous subcutaneous infusions of apomorphine: results of a two‐year, prospective follow‐up. Mov Disord 2002;17:188–191. 11835461 10.1002/mds.1276

[59] Katzenschlager R , Hughes A , Evans A , et al. Continuous subcutaneous apomorphine therapy improves dyskinesias in Parkinson's disease: a prospective study using single‐dose challenges. Mov Disord 2005;20:151–157. 15390035 10.1002/mds.20276

[60] De Gaspari D , Siri C , Landi A , et al. Clinical and neuropsychological follow up at 12 months in patients with complicated Parkinson's disease treated with subcutaneous apomorphine infusion or deep brain stimulation of the subthalamic nucleus. J Neurol Neurosurg Psychiatry 2006;77:450–453. 16543520 10.1136/jnnp.2005.078659 PMC2077512

[61] Antonini A , Isaias IU , Rodolfi G , et al. A 5‐year prospective assessment of advanced Parkinson disease patients treated with subcutaneous apomorphine infusion or deep brain stimulation. J Neurol 2011;258:579–585. 20972684 10.1007/s00415-010-5793-z

[62] Stiggelbout AM , Van der Weijden T , De Wit MP , et al. Shared decision making: really putting patients at the centre of healthcare. BMJ 2012;344:e256. 22286508 10.1136/bmj.e256

[63] Grosset KA , Grosset DG . Patient‐perceived involvement and satisfaction in Parkinson's disease: effect on therapy decisions and quality of life. Mov Disord 2005;20:616–619. 15719417 10.1002/mds.20393

[64] van der Eijk M , Faber MJ , Al Shamma S , Munneke M , Bloem BR . Moving towards patient‐centered healthcare for patients with Parkinson's disease. Parkinsonism Relat Disord 2011;17:360–364. 21396874 10.1016/j.parkreldis.2011.02.012

[65] Page P . Beyond statistical significance: clinical interpretation of rehabilitation research literature. Int J Sports Phys Ther 2014;9:726–736. 25328834 PMC4197528

[66] Shrier I , Boivin JF , Platt RW , et al. The interpretation of systematic reviews with meta‐analyses: an objective or subjective process? BMC Med Inform Decis Mak 2008;8:19. 18495019 10.1186/1472-6947-8-19 PMC2408567

[67] McGlothlin AE , Lewis RJ . Minimal clinically important difference: defining what really matters to patients. JAMA 2014;312:1342–1343. 25268441 10.1001/jama.2014.13128

[68] Trevena LJ , Davey HM , Barratt A , Butow P , Caldwell P . A systematic review on communicating with patients about evidence. J Eval Clin Pract 2006;12:13–23. 16422776 10.1111/j.1365-2753.2005.00596.x

[69] Schunemann HJ , Best D , Vist G , Oxman AD , GRADE Working Group . Letters, numbers, symbols and words: how to communicate grades of evidence and recommendations. CMAJ 2003;169:677–680. 14517128 PMC202287

[70] Trevena LJ , Zikmund‐Fisher BJ , Edwards A , et al. Presenting quantitative information about decision outcomes: a risk communication primer for patient decision aid developers. BMC Med Inform Decis Mak 2013;13(Suppl 2):S7. 10.1186/1472-6947-13-S2-S7 PMC4045391 24625237

[71] Rogers G , Davies D , Pink J , Cooper P . Parkinson's disease: summary of updated NICE guidance. BMJ 2017;358:j1951. 28751362 10.1136/bmj.j1951

[72] Limousin P , Foltynie T . Long‐term outcomes of deep brain stimulation in Parkinson disease. Nat Rev Neurol 2019;15:234–242. 30778210 10.1038/s41582-019-0145-9

[73] Wenzel K , Homann CN , Fabbrini G , Colosimo C . The role of subcutaneous infusion of apomorphine in Parkinson's disease. Expert Rev Neurother 2014;14:833–843. 24917215 10.1586/14737175.2014.928202

[74] Espay AJ , Hausdorff JM , Sanchez‐Ferro A , et al. A roadmap for implementation of patient‐centered digital outcome measures in Parkinson's disease obtained using mobile health technologies. Mov Disord 2019;34:657–663. 30901495 10.1002/mds.27671 PMC6520192

[75] Vizcarra JA , Sanchez‐Ferro A , Maetzler W , et al. The Parkinson's disease e‐diary: developing a clinical and research tool for the digital age. Mov Disord 2019;34:676–681. 30901492 10.1002/mds.27673 PMC8114172

