# Supplementary material for: Translating Evidence to Advanced Parkinson's Disease Patients: A Systematic Review and Meta‐Analysis
Source: Mov Disord. 2021 Apr 2;36(6):1293–307. doi: 10.1002/mds.28599 (PMC8252410; doi:10.1002/mds.28599)
Supplement: Supplementary file 1 — Appendix S1. Supplementary Information [file MDS-36-1293-s001.zip › mds28599-sup-0001-Supinfo/supplement_tables3-5.docx]

**Table S3. Summary of Findings of DBS versus BMT**

| **Certainty assessment** | | | | | | | | **№ of patients** | | **Effect** | | **Certainty** | **Importance** |
| --- | --- | --- | --- | --- | --- | --- | --- | --- | --- | --- | --- | --- | --- |
| **No of studies** | | **Study design** | **Risk of bias** | **Inconsistency** | **Indirectness** | **Imprecision** | **Other considerations** | **DBS** | **BMT** |  | **Absolute (95% CI)** |  |  |
| **Quality of Life (follow up: range 6 months to 24 months; assessed with: PDQ-39)** | | | | | | | | | | | | | |
| 5 | randomized trials | | not serious^a^ | not serious | not serious | not serious | none | 486 | 494 | - | MD **7.4 points lower** (9.3 lower to 5.6 lower) | ⨁⨁⨁⨁ HIGH | CRITICAL |
| **Activities of daily living (follow up: range 6-24 months to 0; assessed with: UPDRS II)** | | | | | | | | | | | | | |
| 5 | randomized trials | | not serious^a^ | serious^b^ | not serious | not serious | none | 468 | 481 | - | MD **1.9 lower** (3.8 lower to 0.0 lower) | ⨁⨁⨁◯ MODERATE | CRITICAL |
| **ON time (follow up: range 6 months to 24 months; assessed with: motor diary, TRS)** | | | | | | | | | | | | | |
| 3 | randomized trials | | not serious ^a^ | serious^c^ | not serious | not serious | none | 279 | 305 | - | MD **3.8 hours higher** (2.0 higher to 5.6 higher) | ⨁⨁⨁◯ MODERATE | CRITICAL |
| **OFF time (follow up: range 6 months to 24 months; assessed with: motor diary, TRS)** | | | | | | | | | | | | | |
| 3 | randomized trials | | not serious ^a^ | serious^b^ | not serious | not serious | none | 280 | 305 | - | MD **2.8 hours lower** (4.2 lower to 1.4 lower) | ⨁⨁⨁◯ MODERATE | IMPORTANT |
| **Serious adverse events (follow up: range 6 months to 24 months; assessed with: number of participants with SAE)** | | | | | | | | | | | | | |
| 4 | randomized trials | | not serious | serious ^b^ | not serious | not serious | none | 506 | 522 | RR:2.31 (1.25;4.28) | **20 patients more with SAE** | ⨁⨁⨁◯ MODERATE | IMPORTANT |

**CI:** Confidence interval; **MD:** Mean difference

**Explanations to downgrading**

a. Though in the studies patients and clinicians were not blinded, this can be explained by the type of intervention. We did not consider the risk of placebo effect that high to downgrade the risk of bias as the design of the RCT were of good quality

b. The heterogeneity test shows a p value of p<0.0001 and the I squared test shows a substantial heterogeneity. The confidence intervals do not fully overlap but the inconsistency is not that large that it must be scored as -2.

c. The heterogeneity test shows a p value <0.05 and I squared test shows substantial heterogeneity, however the outlier is the study of Shuepbach therefore scored as -1 and not as -2, (heterogeneity is known as these patients are patients with early motor complications and the other studies have patients with late motor complications (longer PD duration) )

**Table 4. Summary of findings of LCIG versus BMT**

| **Certainty assessment** | | | | | | | **№ of patients** | | **Effect** | | **Certainty** | **Importance** |
| --- | --- | --- | --- | --- | --- | --- | --- | --- | --- | --- | --- | --- |
| **№ of studies** | **Study design** | **Risk of bias** | **Inconsistency** | **Indirectness** | **Imprecision** | **Other considerations** | **LCIG** | **BMT** | **Relative (95% CI)** | **Absolute (95% CI)** |  |  |
| **Quality of life (follow up: range 6 weeks to 12 weeks; assessed with: PDQ-39)** | | | | | | | | | | | | |
| 2 ^a^ | randomized trials | not serious^b^ | not serious | not serious | serious ^c^ | none | 35 | 31 | - | MD **7 points lower** (16 lower to 2 higher) | ⨁⨁⨁◯ MODERATE | CRITICAL |
| **Quality of Life (follow up: range 6 months to 1 years; assessed with: PDQ39, PDQ8)** | | | | | | | | | | | | |
| 6 | observational studies | very serious ^d^ | very serious ^e^ | not serious | serious^f^ | none | 482 |  | - | MD **16.9 points lower** (31.1 lower to 2.7 lower) | ⨁◯◯◯ VERY LOW | CRITICAL |
| **Quality of Life (follow up: range 2 years to 3 years; assessed with: PDQ-39, PDQ-8)** | | | | | | | | | | | | |
| 5 | observational studies | very serious ^d^ | serious^g^ | not serious | not serious | none | 257 |  | - | **MD 11.3 points lower** (16.7 lower to 6.0 lower) | ⨁⨁◯◯ LOW | CRITICAL |
| **Activities of daily living (follow up: mean 12 weeks; assessed with: UPDRS II)** | | | | | | | | | | | | |
| 2 ^a^ | randomized trials | not serious^b^ | not serious | not serious | serious^c^ | none | 35 | 31 | - | MD **3.0 points lower** (5.3 lower to 0.8 lower) | ⨁⨁⨁◯ MODERATE | CRITICAL |
| **Activities of Daily Living (follow up: 12 months; assessed with: UPDRS II)** | | | | | | | | | | | | |
| 6 | observational studies | serious^d^ | not serious | not serious | not serious | none | 457 |  | - | MD **4.0 points lower** (4.6 lower to 3.4 lower) | ⨁◯◯◯ VERY LOW | CRITICAL |
| **Activities of Daily Living (follow up: range 2 years to 3 years; assessed with: UPDRS II)** | | | | | | | | | | | | |
| 5 | observational studies | serious^a^ | serious^b^ | not serious | serious^f^ | none | 239 |  | - | MD **0.3 points higher** (2.6 lower to 3.1 higher) | ⨁◯◯◯ VERY LOW | CRITICAL |
| **ON time (follow-up 4 days- 12 weeks; assessed with motor diary)** | | | | | | | | | | | | |
| 2^h^ | randomized trials | not serious^h^ | not serious^i^ | not serious | not serious | none | 35 | 31 | - | MD **1.9 hours higher** ( 0.6 higher to 3.2 higher) | ⨁⨁⨁◯ MODERATE^j^ | CRITICAL |
| **ON time (follow up: 54 weeks; assessed with motor diary)** | | | | | | | | | | | | |
| 1 | observational studies | serious^k^ | not serious | not serious | not serious | none | 307 |  | - | MD **4.8 hours higher** (4.4 higher to 5.2 higher) | ⨁◯◯◯ VERY LOW | CRITICAL |
| **OFF time (follow up: 4 days -12 weeks; assessed with motor diary)** | | | | | | | | | | | | |
| 2^i^ | randomized trials | not serious^h^ | not serious^i^ | not serious | not serious | none | 35 | 31 | - | MD **1.9 hours lower** (3.1 lower to 0.8 lower) | ⨁⨁⨁◯ MODERATE^j^ | IMPORTANT |
| **OFF time (follow up: 12 months; assessed with: motor diary)** | | | | | | | | | | | | |
| 3 | observational studies | very serious^d^ | not serious | not serious | not serious | none | 399 |  | - | MD **4.9 hours lower** (5.7 lower to 4.1 lower) | ⨁◯◯◯ VERY LOW | IMPORTANT |
| **Serious adverse events (follow up: range 6 weeks to 12 weeks; assessed with: number of participants with SAE)** | | | | | | | | | | | | |
| 2^l^ | randomized trials | not serious^l^ | not serious^i^ | not serious | serious^m^ | Patients in BMT group also had a PEG-J tube placement | 37 | 34 | **RR 0.66** (0.23 to 1.87) | **7 fewer per 100 patients** | ⨁⨁◯◯ LOW | IMPORTANT |
|  |  |  |  |  |  |  | - | 0.0% |  |  |  |  |
| **Serious adverse events (assessed with: number of participants with SAE)** | | | | | | | | | | | | |
| 8 | observational studies | serious^d^ | serious^n^ | not serious | not serious | none | 644 participants |  |  | 17 per 100 patients  ( 9-31) in LCIG group with a SAE | ⨁⨁◯◯ LOW | IMPORTANT |
|  |  |  |  |  |  |  | - |  |  | Not known for BMT |  |  |

**CI:** Confidence interval; **MD:** Mean difference; **RR:** Risk ratio **SAE**: Serious adverse event

**Explanations to downgrading**

a. One RCT has no baseline data available for this outcome, only median effect given between LCIG en BMT, therefore not included in calculation effect

b. Blinding was not done in one study ( study for calculation effect was blinded). Intervention is difficult to mask; therefore, we did not deduct a point for this. It was unclear if there was selective reporting, this was not considered substantial, therefore no point was deducted

c. Only one study for calculation with relatively small group of participants and large confidence interval, which includes the minimal important difference (but cohort studies show similar effect)

d. High risk for selection bias, high risk for performance bias, high risk for detection bias, high risk for attrition bias, unclear risk for selective reporting bias

e. Test for heterogeneity (I2) is very high, confidence intervals overlap but with one clear outlier

f. Large confidence intervals which includes the MCID

g. Test for heterogeneity is very high, confidence intervals overlap, no outliers

h. One RCT is a crossover trial and used a different scale, for cross-over trial unclear how randomized, and high risk other bias( very short washout period, Very short follow up duration in crossover trial) therefore calculation effect only given for one study and risk of bias for that study displayed

i. If both studies combined it would show serious inconsistency

j. If both studies combined serious inconsistency and risk of bias (due to other reasons only one RCT chosen for calculation effect), one study with low risk of bias and small sample size therefore graded as moderate certainty

k. High risk for selection bias, high risk performance bias, high risk detection bias, low risk attrition bias, low risk selective reporting bias

l. One study has used nasoduodenal tube which excludes operation risks ( does not represent daily practice) therefore calculation effect based on one study ( excluding crossover trial with nasoduodenal tube). Blinding was not done in one study ( study for calculation effect was blinded). Intervention is difficult to mask; therefore, we did not deduct a point for this. It was unclear if there was selective reporting, this was not considered substantial, therefore no point was deducted. High risk other bias in one study( short washout period).

m. Large confidence intervals

n. The heterogeneity test shows a p value <0.05 and I squared test shows substantial heterogeneity

**Table 5. Summary of Findings CSAI versus BMT**

| **Certainty assessment** | | | | | | | **№ of patients** | | **Effect** | | **Certainty** | **Importance** |
| --- | --- | --- | --- | --- | --- | --- | --- | --- | --- | --- | --- | --- |
| **№ of studies** | **Study design** | **Risk of bias** | **Inconsistency** | **Indirectness** | **Imprecision** | **Other considerations** | **CSAI** | **BMT** | **Relative (95% CI)** | **Absolute (95% CI)** |  |  |
| **Quality of Life (follow up: 12 weeks; assessed with: PDQ8)** | | | | | | | | | | | | |
| 1 | randomized trials | not serious | not serious | not serious | very serious^a^ | none | 53 | 53 | - | MD **2.5 points lower** (7.6 lower to 2.7 higher) | ⨁⨁◯◯ LOW | CRITICAL |
| **Quality of Life (follow up: mean 12.5 months; assessed with: PDQ-8)** | | | | | | | | | | | | |
| 1 | observational studies | serious ^b^ | not serious | not serious | serious ^c^ | Large effect | 17 | 17 | - | MD **32.2 points lower** (47.7 lower to 16.6 lower) | ⨁◯◯◯ VERY LOW | CRITICAL |
| **Quality of life (follow up: mean 6 months; assessed with: PDQ-8)** | | | | | | | | | | | | |
| 1 | observational studies | very serious ^d^ | not serious | not serious | not serious | none | 43 |  | - | MD **14.8 points lower** (20.0 lower to 9.6 lower) | ⨁⨁◯◯ LOW | CRITICAL |
| **Activities of daily living (follow up: range 6 months to 66 months; assessed with: UPDRS II, S&E, L&F)** | | | | | | | | | | | | |
| 3 ^e^ | observational studies | very serious ^f^ |  | very serious ^e^ |  | none | 0 | 0 | - | NA | - | CRITICAL |
| **ON time (follow up: range 6-12 months to 0; assessed with: hours per day)** | | | | | | | | | | | | |
| 1 | randomized trials | not serious | not serious | not serious | serious ^g^ | none | 53 | 53 | - | MD **2.0 hours higher** (0.7 higher to 3.2 higher) | ⨁⨁⨁◯ MODERATE | CRITICAL |
| **ON time (follow up: 6 months; assessed with: Percentage ON time per day/diary)** | | | | | | | | | | | | |
| 1 | observational studies | serious ^h^ | not serious | not serious | serious ^i^ | none | 12 |  | - | MD **20 percent higher** (0 to 0) | ⨁◯◯◯ VERY LOW | CRITICAL |
| **OFF time (follow up: 12 weeks; assessed with: hours per day)** | | | | | | | | | | | | |
| 1 | randomized trials | not serious | not serious | not serious | serious ^g^ | none | 53 | 53 | - | MD **1.9 hours lower** (3.2 lower to 0.6 lower) | ⨁⨁⨁◯ MODERATE | IMPORTANT |
| **OFF time (follow up: mean 1 years; assessed with: hours per day)** | | | | | | | | | | | | |
| 1 | non-randomized studies | serious ^j^ | not serious | not serious | not serious | none | 12 | 18 | - | MD **3.5 hours lower** (4.7 lower to 2.3 lower) | ⨁⨁◯◯ LOW | IMPORTANT |
| **OFF time (follow up: range 6 months to 12 months; assessed with: hours OFF time per day, percentage OFF hours per day, percentage change OFF time)** | | | | | | | | | | | | |
| 3 | observational studies ^k^ | very serious ^l^ | serious ^m^ | not serious | serious ^g^ | none | 36 |  | - | MD **3.3 hours lower** (6.4 lower to 0.3 lower) | ⨁◯◯◯ VERY LOW | IMPORTANT |
| **serious adverse events (follow up: 12 weeks; assessed with: number of participants with serious adverse event)** | | | | | | | | | | | | |
| 1 | randomized trials | not serious | not serious | not serious | serious ^c^ | none | 53 | 53 | **RR 2.45** (0.5 to 12.10) | **5 more persons** **per 100** | ⨁⨁⨁◯ MODERATE | CRITICAL |
| **serious adverse events (follow up: mean 12 months; assessed with: number of persons with SAE)** | | | | | | | | | | | | |
| 2 | observational studies | serious ^h^ | not serious | not serious | serious ^c^ | none | 56 |  | - | **3 persons per 100** (0 to 71) in CSAI group  BMT not known | ⨁◯◯◯ VERY LOW | CRITICAL |

**CI:** Confidence interval; **MD:** Mean difference; **RR:** Risk ratio

#### Explanations to downgrading

a. Downgraded to very serious limitation due to large confidence interval and includes no effect

b. Unclear risk selection bias, high risk allocation concealment, high risk blinding (inevitable because of intervention bias, not downgraded), low risk attrition bias, low risk reporting bias

c. Large confidence intervals

d. High risk for selection bias, high risk for blinding (inevitable because of intervention nature, not downgraded), high risk for attrition bias, low risk for selective reporting bias and low risk for other bias)

e. Due to use of different scales and measure methods not possible to pool data

f. High risk for selection bias, high risk for performance bias and high risk for detection bias, 1 study had low risk for attrition bias, 2 studies unclear risk and 1 study high risk attrition bias, 2 studies low risk and 2 studies unclear risk on reporting bias, 2 studies high risk other bias due to very small sample size and one study had high risk for other bias due to a 2 month trial period before patients were included in the analysis

g. Downrated for imprecision because confidence interval includes the minimal important difference

h. High risk selection bias, high risk performance bias, high risk detection bias, low risk attrition bias, low risk reporting bias

i. Small number of patients, confidence interval not known

j. High risk for selection bias, high risk for blinding (inevitable because of intervention nature, not downgraded), low risk attrition bias, unclear risk reporting bias, unclear risk other bias due to low quality of study design and reporting

k. Case-control and other study designs together

l. High risk for selection bias, high risk for blinding (inevitable because of intervention nature, not downgraded), 3 studies have an unclear risk for attrition bias, 2 studies unclear risk for selective reporting bias and 1 study has a low risk for selective reporting bias, all studies have a high risk for other bias because of very small sample size

m. One study shows a much larger effect than the others while in 2 studies the confidence intervals include the minimal important difference
